# Author Correction: Dynamically induced cascading failures in power grids

**DOI:** 10.1038/s41467-018-06483-9

**Published:** 2018-09-27

**Authors:** Benjamin Schäfer, Dirk Witthaut, Marc Timme, Vito Latora

**Affiliations:** 1 Chair for Network Dynamics, Center for Advancing Electronics Dresden (cfaed) and Institute for Theoretical Physics, Technical University of Dresden, 01062 Dresden, Germany; 2 Network Dynamics, Max Planck Institute for Dynamics and Self-Organization (MPIDS), 37077 Göttingen, Germany; 3 Forschungszentrum Jülich, Institute for Energy and Climate Research - Systems Analysis and Technology Evaluation (IEK-STE), 52428 Jülich, Germany; 4 Institute for Theoretical Physics, University of Cologne, 50937 Köln, Germany; 5 School of Mathematical Sciences, Queen Mary University of London, London, E1 4NS UK; 6 Dipartimento di Fisica ed Astronomia, Università di Catania and INFN, 95123 Catania, Italy

Correction to: *Nature Communications* 10.1038/s41467-018-04287-5; published online 17 May 2018

The original version of this Article omitted the following from the Acknowledgements:

‘Finally, we gratefully acknowledge support from the German Science Foundation (DFG) by a grant toward the Cluster of Excellence “Center for Advancing Electronics Dresden” (cfaed)’

This has been corrected in both the PDF and HTML versions of the Article.

