# Supplementary material for: Dynamically induced cascading failures in power grids
Source: Nat Commun. 2018 May 17;9:1975. doi: 10.1038/s41467-018-04287-5 (PMC5958123; doi:10.1038/s41467-018-04287-5)
Supplement: Supplementary file 1 — Supplementary Information [file 41467_2018_4287_MOESM1_ESM.pdf]

Supplementary Information
accompanying the manuscript
Dynamically induced cascading failures in power grids
by

Benjamin Schäfer,[1,2] Dirk Witthaut,[3,4] Marc Timme,[1,2] and Vito Latora[5,6]

[1] *Chair for Network Dynamics, Center for Advancing Electronics Dresden (cfaed) and Institute for Theoretical Physics, Technical University of Dresden, 01062 Dresden, Germany*

[2] *Network Dynamics, Max Planck Institute for Dynamics and Self-Organization (MPIDS), 37077 Göttingen, Germany*

[3] *Forschungszentrum Jülich, Institute for Energy and Climate Research - Systems Analysis and Technology Evaluation (IEK-STE), 52428 Jülich, Germany*

[4] *Institute for Theoretical Physics, University of Cologne, 50937 Köln, Germany*

[5] *School of Mathematical Sciences, Queen Mary University of London, London E1 4NS, United Kingdom*

[6] *Dipartimento di Fisica ed Astronomia, Università di Catania and INFN, I-95123 Catania, Italy*

This Supplementary Information follows the general narrative of the main manuscript, adding to it more detailed descriptions of adopted methods and of the results obtained for different networks and models. In particular, it includes a technical description of the cascade implementation, plots and results referring to network topologies of Great Britain, Spain and France and formulas to calculate the Line Outage Distribution Factor (LODF). Furthermore, we show that results presented in the main text do not change qualitatively when we investigate these different grid topologies, or when we adopt different models for the power grid flow including a third order model with voltage dynamics and power flow computations that also compute reactive power flows and ohmic losses. Finally, we investigate the propagation of the cascade and we compare results obtained by using the effective graph distance, with those obtained with a standard measure of graph distance.

SUPPLEMENTARY NOTE 1

Basic methods

Here, we describe the basic methods to simulate and analyze cascading failures. Namely, we provide additional technical details that were used to produce our results, give definitions for the number of unsynchronized nodes, discuss our choice of test grids and present the computation of Line Outage Distribution Factors (LODF).

Implementation of cascading failures

Motivated by the short time scale of cascading failures in the real world [1–3], we model the flows dynamically. To this end, we consider the swing equation [4, 5] given by:

$$\frac{\mathrm{d}}{\mathrm{d}t}\theta_i = \omega_i, \tag{1}$$

$$\frac{\mathrm{d}}{\mathrm{d}t}\omega_i = P_i - \gamma\omega_i + \sum_{j=1}^{N} K_{ij}\sin\left(\theta_j - \theta_i\right), \tag{2}$$

where $\theta_i(t)$ represents the mechanical rotor angle at node i at time t, and $\omega_i(t)$ is the angular velocity. P_i is the active power at a node, γ a damping constant, which we assume to be homogeneous, and K_{ij} gives the coupling strength between two connected nodes. In order to analyze cascades, we numerically solve this set of coupled nonlinear differential equations for $i = 1, 2, \ldots, N$. Each simulation is started at the fixed point (θ_i^*, ω_i^*), which is defined, for a given topology of the power network, as the solution of the equations:

$$\omega_i^* = 0, \tag{3}$$

$$P_i + \sum_{j=1}^{N} K_{ij}\sin\left(\theta_j^* - \theta_i^*\right) = 0, \tag{4}$$

for $i = 1, 2, \ldots, N$. Due to the nonlinearity of the equations, the fixed point angles θ_i^* cannot be expressed in a closed form. Notice that in general the fixed point of this set of equations is not unique, but multiple fixed points may exist [6]. However, as long as the (homogeneous) coupling K is close to the critical coupling, $K \sim K_c$, where K_c is the minimal coupling for a fixed point to exist, there is only one fixed point [7]. We determine the fixed point of the power grid using Newton's method. This is done by starting with an initial guess $\theta_{i0}^* = 0$ and $\omega_{i0}^* = 0$ for all $i \in \{1, ..., N\}$. Next, we let Newton's method converge to an actual fixed point solution for Supplementary Equations (3)-(4). Then, we start the numerical simulation at this fixed point , i.e., we set the initial conditions as:

$$\omega_i(t=0) = 0,$$
$$\theta_i\,(t=0) = \theta_i^*,$$

and we wait until the trigger time $t_{\text{trigger}} = 1s$ to cut one line (deactivate one link) of the power grid, which we call the trigger line. If cutting the line changes the fixed point, a transient dynamic towards the new fixed point sets in, otherwise the simulation terminates.

We then assume that real power grids are never operated at their absolute physical limit, but that security margins will cause lines to shut down only if they exceed a critical value of the flow [4, 8]. In practice, we implement the following rule. The additional line (i, j) fails and is cut if the flow along such a line, defined as:

$$F_{ij}\,(t) = K_{ij}\sin\left(\theta_j\,(t) - \theta_i\,(t)\right), \tag{5}$$

exceeds the capacity of the line, $C_{ij} = C_{ij}\,(\alpha)$, which depends on a tolerance parameter α :

$$F_{ij} > C_{ij}\,(\alpha) = \alpha K_{ij}, \tag{6}$$

where the tolerance parameter can be at most one, namely $\alpha \leq 1$. This procedure is different from those adopted in other works on cascade, which use instead a threshold dependent on the initial flow in the network [9–11]. However, it seems much more appropriate for power grids where the threshold at which a line has to be shut down does not depend on its initial load, but on its physical capacity [12]. Note that in our model the flow is changing over time and gets influenced by additional line failures, as flows from other parts of the networks will get re-routed. We continue to track the flow and the failure of overloaded lines by using an event-detector in our ODE solver [13] until a maximum time $t_{max} = 50\,\mathrm{s}$, at which, in all cases considered, no more lines fail and the cascade is finished.

Definition of unsynchronized nodes

Besides the information which flow F_{ij} exceeds the threshold, and hence which lines get overloaded, we also record the final number of unsynchronized nodes after the cascade of failures is over. The definition of unsynchronized node we adopted is based on the assumption that a frequency deviation of $\Delta f \sim 20\,\mathrm{mHz}$ is well within the stable operation boundaries of the European grid [4, 14]. Consequently, a node i is recorded as unsynchronized if:

$$|\omega_i\,(t_{\max})| > 2\pi\,0.02\ \mathrm{Hz},$$

i.e., if its angular velocity at the end of the simulation, namely at time $t_{\max} = 50\,s$, is larger than the adopted threshold. The nodes showing large deviations from the reference frequency would most likely have to be disconnected from the grid, e.g. via load shedding [4]. Thereby, the number of unsynchronized nodes in a network is a good proxy for the number of affected consumers. In our case, the comparably strict choice of the threshold $\Delta f = 20\,\mathrm{mHz}$ was chosen to ensure that the system is at a fixed point and not on a limit cycle with small amplitude.

Test grids

Dynamical cascades were mainly investigated in networks based on the real structure of the high voltage transmission grids of Spain, France [15] and Great Britain [16, 17].

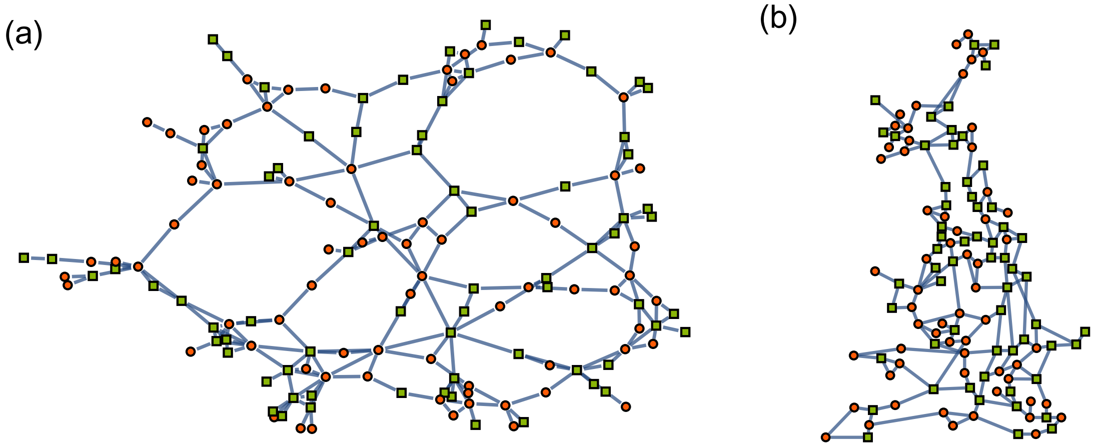

Supplementary Figure 1. In addition to the Spanish topology presented in the main text, we have also studied cascading failures in the real power grid topologies of France [15] and Great Britain [16, 17]. (a) The French grid has a clustering coefficient smaller than that of the Spanish grid. (b) The grid of Great Britain has an even smaller clustering coefficient, but many 4-cycles. We display both networks with a set of distributed generators (green squares), with $P^{+} = 1/s^2$ and consumers (red circles), with $P^{-} = -1/s^2$. The network topologies are available online, see data availability statement.

Results for the number of line failures for the Spanish and French grid are discussed in the main text, while in this Supplementary Information we report results on the number line failures in the British grid, and on the number of unsynchronized nodes in all three grids. The topology of the high voltage transmission grid of Spain is shown in the main text, while the networks of France and Great Britain, with randomly distributed generators and consumers are displayed in Supplementary Fig. 1. All grids are considered both with distributed small generator nodes with $P^{+} = 1$ (Supplementary Fig. 1) as well as fewer and large generator nodes with $P^{+} \approx 6$ (not shown, see Data Availability Statement for network topologies), i.e, each large generator is supplying approximately six consumer nodes.

Finally, we also considered heterogeneous coupling, where the capacity of each line is chosen so that the line is approximately loaded to 50%. To construct a heterogeneous coupling matrix K_{ij}, we use an iterative procedure that adapts the capacity to the flow. The grid is initialized with distributed generators and homogeneous coupling $K_{ij}^{\mathrm{old}} = K$, where the constant K has been set to $K = 8/s^2$ for the French topology, and $K = 5/s^2$ for both the Spanish and the British topologies. Next, the initial load on each line (i, j) is computed, and the new coupling is set to:

$$K_{ij}^{\mathrm{new}} = 0.99 K_{ij}^{\mathrm{old}} + 0.01 K_{ij}^{\mathrm{old}} F_{ij}^{\mathrm{old}}/0.5. \tag{7}$$

Finally, we set $K_{ij}^{\text{old}} = K_{ij}^{\text{new}}$ for all links, and the next fixed point is computed together with the associated flows F_{ij}^{old}. The procedure is repeated for a total of 200 times. In this way the network approaches a state where every line is loaded to about 50% of its physical maximum, namely $F_{ij} \approx 0.5K_{ij}$ for all links (i,j).

Computing the Line Outage Distribution Factor (LDF)

In order to save computational time when determining fixed points and hence the new steady-state flows, we use the Line Outage Distribution Factor (LODF) [18, 19]. The LODF approximates line flows after the trigger link (a,b) is removed as:

$$F_{ab} = 0, \tag{8}$$

$$F_{ij}^{\text{new}} \approx F_{ij}^{\text{old}} - F_{ab}^{\text{old}} \frac{\tilde{K}_{ij}\left(T_{ja} - T_{jb} - T_{ia} + T_{ib}\right)}{1 - \tilde{K}_{ab}\left(T_{aa} - T_{ab} - T_{ba} + T_{bb}\right)}, \tag{9}$$

where a, b are the labels of the trigger line, and i, j are the labels of any other line. In the expressions above we make use of two auxiliary matrices, namely $\tilde{\mathbf{K}} = \{\tilde{K}_{ij}\}$ and $\mathbf{T} = \{T_{ij}\}$. The first one is defined as:

$$\tilde{K}_{ij} = K_{ij} \cos\left(\theta_i^* - \theta_j^*\right), \tag{10}$$

where θ_i^*, $i = 1, 2, \ldots, N$, are the fixed point angles of the intact network, while the auxiliary matrix T is the Moore-Penrose pseudoinverse of matrix $\mathbf{A}$ given by:

$$A_{ij} = \begin{cases} -\tilde{K}_{ij} & \text{for } i \neq j \\ \sum_l \tilde{K}_{lj} & \text{for } i = j \end{cases}, \tag{11}$$

In the main text, we made use of these approximated flows to detect the critical lines to be deactivated.

SUPPLEMENTARY NOTE 2

Analysis of the British grid and of unsynchronized nodes

Results on the number of line failures in the Spanish and French grids were reported and discussed in the main text. Here, we repeat the same type of analysis for the British power grid, and we also show that our dynamical flow-based predictor performs better than other predictors in the case of the British power grid. But first we present results on the number of unsynchronized nodes (as a measure of how many customers would be affected by a blackout) after the cascade terminates.

Number of unsynchronized nodes

In the main text we have investigated the statistics of line failures for different grids, and we noticed that most trigger lines cause no additional cascade or very small cascades. Supplementary Fig. 2 reports the corresponding statistics for the number of unsynchronized nodes. We observe a very similar behavior, i.e., either the whole grid is affected by the initial failure of a line, i.e., nearly all nodes lose synchrony, or nothing happens and the grid maintains its steady state. Interestingly, we observe that this all-or-nothing response is more pronounced in the case of homogeneous coupling (distributed and centralized power), while heterogeneous couplings allow for more intermediate situations. This is opposite to what was observed in the main text for the number of line failures, where homogeneous coupling resulted instead in broader distributions. However, the key message is unchanged: only a few critical initial triggers can cause large cascades. Hence, it can be very useful to be able to identify such initial triggers, as we did in the main text.

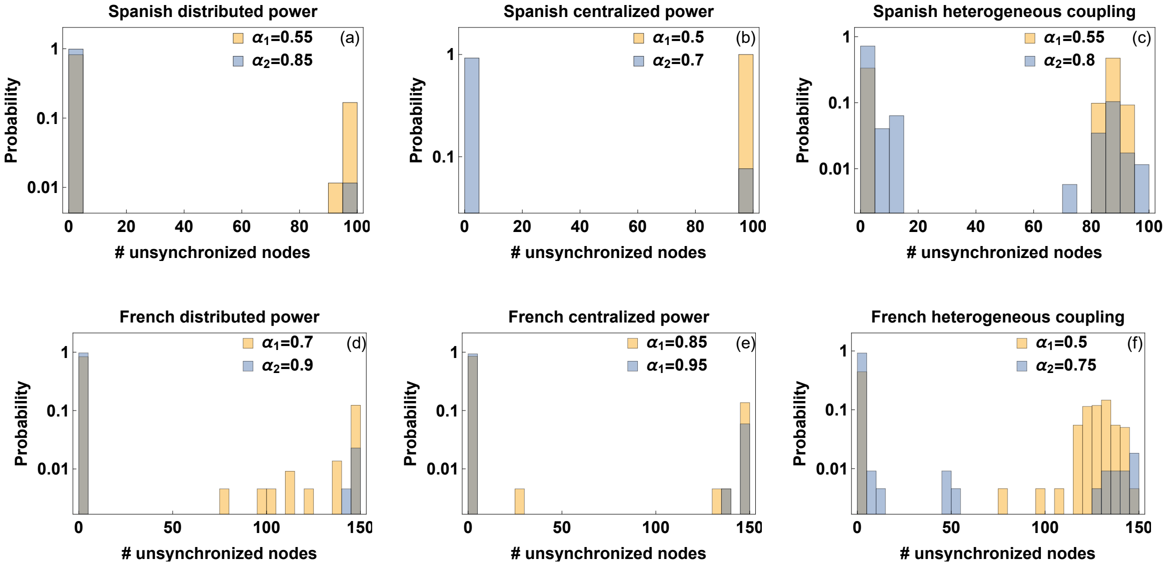

Supplementary Figure 2. Node desynchronization probability in the Spanish and French power grids under different power distributions and types of coupling. The histograms shown have been obtained under three different settings, see also main text. Panels (a) and (d) refer to the case of *distributed power*, i.e., equal number of generators and consumers, each with $P^{+} = 1/s^2$ and $P^{-} = -1/s^2$, and homogeneous coupling with $K = 5/s^2$ for the Spanish and $K = 8/s^2$ for the French grid. Panels (b) and (e) refer to the case of *centralized* power, i.e., consumers with $P^{-} = -1/s^2$ and fewer but larger generators with $P^{+} \approx 6/s^2$, and homogeneous coupling with $K = 10/s^2$ for Spanish and $K = 9/s^2$ for the French grid. Panels (c) and (f) refer to a case of distributed power as in panel (a) and (d), but with *heterogeneous coupling*, so that the fixed point flows on the lines are approximately $F \approx 0.5K$ both for the Spanish and the French grid. For all plots we use two different tolerances, where the lower one is the smallest simulated value of α so that there are no initially overloaded lines ($N - 0$ stable).

Cascading failures in the power grid of Great Britain

In the main text we have reported the analysis of the statistical properties of cascades in the Spanish and French power grids. Here, we investigate the case of Great Britain, using the topology of the transmission network shown in Supplementary Fig. 1(b). The histograms in Supplementary Fig. 3 show the probability to observe a given number of line failures and unsynchronized nodes at the end of the cascade. The results are qualitatively similar to those obtained for the other topologies. Most links do cause only small cascades or no cascade at all, especially for homogeneous coupling. On the contrary, a few initial triggering lines can cause large damages, in particular for the heterogeneous coupling case, with tolerance $\alpha_1 = 0.55$, as shown in panel (c).

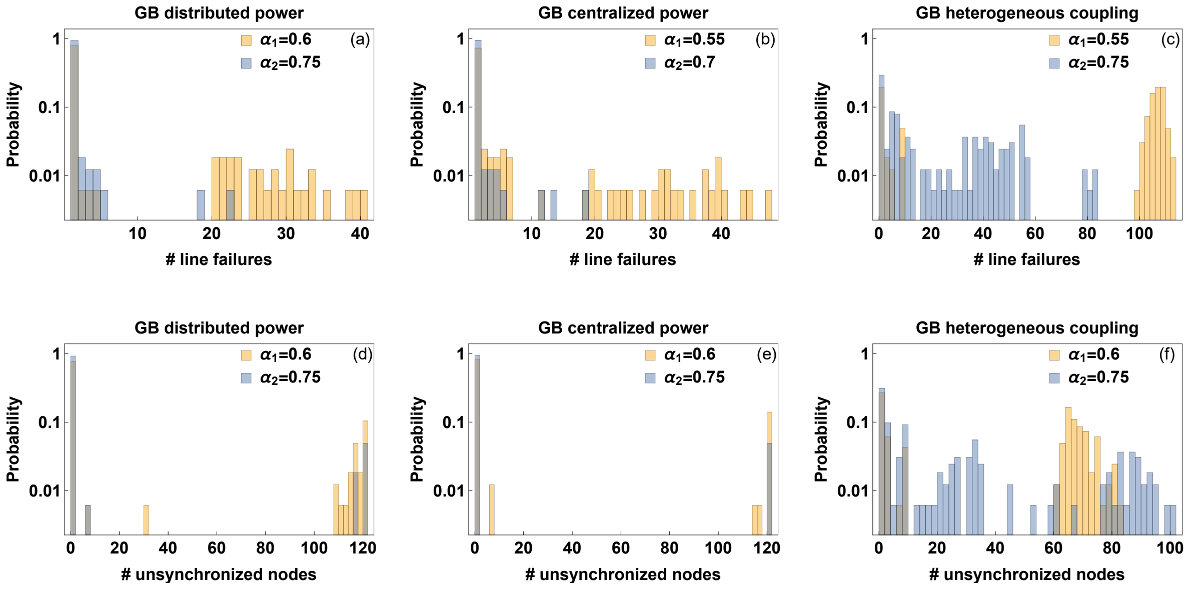

Supplementary Figure 3. Network damage (a-c) and number of unsynchronized nodes (d-f) distributions in the power grid of Great Britain (GB) under different power allocations and types of coupling. (a) and (d) *Distributed power*, i.e., equal number of generators and consumers, each with $P^{+} = 1/s^2$ and $P^{-} = -1/s^2$, and homogeneous coupling with $K = 5/s^2$. (b) and (e) *Centralized* power, i.e. consumers with $P^{-} = -1/s^2$ and fewer but larger generators with $P^{+} \approx 6/s^2$, and homogeneous coupling with $K = 12/s^2$ is investigated. (c) and (f) Same distributed power as in panel (a), but with *heterogeneous coupling*, i.e. coupling on all lines scaled in such a way that all lines are approximately loaded to half of their maximum capacity, namely $F \approx 0.5K$. In all panels we use two different tolerances α, where the lower one is the smallest simulated value of α, so that there are no initially overloaded lines ($N - 0$ stable). The grid has $N_{\mathrm{GB}} = 120$ nodes and $|E|_{\mathrm{GB}} = 165$ edges.

Furthermore, the flow-based predictor introduced in the main text performs very well also on the British topology, as shown in Supplementary Fig. 4. Specifically, it outperforms alternative predictors like those based on the initial load of lines or on the betweenness centrality.

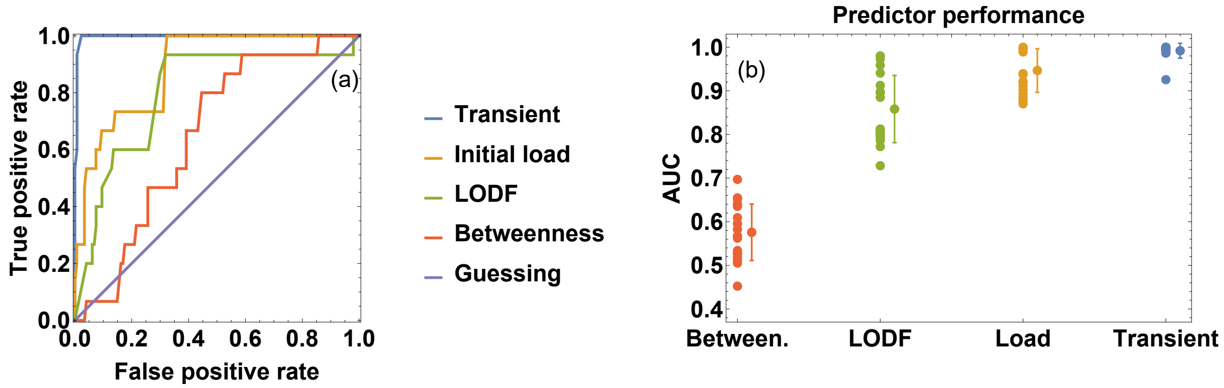

Supplementary Figure 4. Comparing the predictions of our flow-based indicator of critical lines to other standard measures in the case of the British power grid topology. As in the main text, four different predictors are presented to determine whether a given line, if chosen as initially damaged, causes at least one additional line failure. Our dynamical predictor (indicated as Transient) is based on the estimated maximum transient flow. The predictor based on the Line Outage Distribution Factor (LODF) uses the same idea but computes the new fixed flows based on a linearization of the flow computation. Predictors based on betweenness and initial load classify a line as critical if it is within the top $\sigma^{th} \times 100\%$ of the edges with highest betweenness/load with threshold $\sigma^{th} \in [0, 1]$. (a) The predictors are tested against simulations via a Receiver-Operator-Characteristic (ROC) curve recording true positive rate and false positive rate of all predictors for different alarm thresholds. The analysis uses the Great Britain grid with heterogeneous coupling and tolerance $\alpha = 0.6$. (b) The Area Under the (ROC) Curve, AUC, of each predictor is displayed for the Great Britain Grid under different network settings (randomized generator positions, using distributed and centralized power as well as heterogeneous coupling). For each predictor all individual scores are displayed on the left, and the mean with error bars based on one standard deviation is shown on the right. The dynamical flow-based predictor outperforms clearly all other predictors also in the case of the British power grid.

SUPPLEMENTARY NOTE 3

Comparing different models

Although the swing equation is a simplified dynamical model, it allows to capture the essential dynamics of power grid systems [4, 12]. To illustrate this, we compare here the properties of cascading failures emerging in our model with the ones obtained by power flow analysis and by using a third order dynamical model. While power flow analysis is often used to assess the static grid behavior in engineering literature [4, 8, 12], the third order model is an extension of the swing equation where the voltage is dependent on time [4, 20–22]. First, we introduce these two models, and we then compare the cascades obtained by using all models. We conclude with a brief discussion on the validity of the swing equation.

The power flow model

The power flow or load flow equations are a common tool to assess steady state power grid flows in the engineering literature [4, 8, 12]. They assume that the angular velocity is zero $\omega = 0$ because only the steady state is analyzed. The power grid network is characterized by the susceptance matrix $\mathbf{B} = \{B_{ij}\}$ and the conductance matrix $\mathbf{G} = \{G_{ij}\}$, which leads to the following equations for the active power, P_i, and the reactive power, Q_i, at each node i, $i = 1, 2, \ldots, N$:

$$P_i = V_i \sum_{j=1}^{N} \left(G_{ij} V_j \cos(\theta_i - \theta_j) + B_{ij} V_j \sin(\theta_i - \theta_j) \right), \tag{12}$$

$$Q_i = V_i \sum_{j=1}^{N} \left(G_{ij} V_j \sin(\theta_i - \theta_j) - B_{ij} V_j \cos(\theta_i - \theta_j) \right), \tag{13}$$

where θ_i and V_i are respectively the voltage phase angle and the voltage amplitude of node i at equilibrium. Since we have two equations for each node, but four variables, namely θ_i, V_i, Q_i and P_i, we need to have two quantities given as input per node. Depending on which quantities are known and which are unknown, each node (or bus) is characterized as follows. At the so-called *slack (swing) bus*, the voltage amplitude V_i and voltage angle θ_i are specified, while P_i and Q_i are unspecified to compensate power loss in the system. Typically, this would be one of the largest generators that is stabilizing the grid. In addition, there are *voltage-controlled buses (PV)*, which are usually generator nodes for which P_i and V_i are fixed, while we need to solve the equations for Q_i and θ_i. Finally, there exist *load buses (PQ)* with constant active power P_i and reactive power Q_i, but unknown voltage amplitude V_i and voltage angle θ_i [8]. Compared to the swing equations presented in the main text, the power flow equations include the reactive power Q_i of a node, and allow to take into account ohmic losses through the use of the conductance matrix $\mathbf{G}$. However power flow equations only allow comparison of fixed points, since there is no dynamical evolution included in such a modeling.

The third order model

The third order model [4, 20–23] is similar to the swing equation but, in addition to the angle θ_i and the angular velocity ω_i at each node i, it also allows to take into account of the variations over time of the voltage amplitude V_i. The corresponding equations, for $i = 1, 2, \ldots, N$, read:

$$\frac{\mathrm{d}}{\mathrm{d}t}\theta_i = \omega_i \tag{14}$$

$$\frac{\mathrm{d}}{\mathrm{d}t}\omega_i = P_i - \gamma\omega_i + \sum_{j=1}^{N} V_i V_j B_{ij} \sin(\theta_j - \theta_i) \tag{15}$$

$$\frac{\mathrm{d}}{\mathrm{d}t}V = \frac{1}{T_V}\left(V_f - V_i + X \sum_{j=1}^{N} V_j \cos(\theta_j - \theta_i) \right), \tag{16}$$

where P_i is the real power injection at node i, γ is the damping factor (see main text), $T_V = 1/2$ is the voltage time scale, $\mathbf{B} = \{B_{ij}\}$ is the susceptance matrix, which also includes self-coupling terms, B_{ii}. Finally $V_f = 1$ is the

voltage set-point, while X is the voltage droop. For $X = 0$ and $V(t = 0) = 1$ the voltage remains at the fixed point $V^* = 1$ at all times, and the model reduces to the second order model, while for $X > 0$ deviations from the second order model can be observed. Typical parameter values are taken from [20]. Note also that the voltage dynamics is typically slower than the angle and angular velocity dynamics. It can therefore be neglected for short time scales.

Comparison of the effects of cascades

After introducing the power flow and the third order model, let us compare cascades obtained by using these two models with those produced by the the swing equation. In all cases, we simulate a cascade by comparing the sine of the angle difference to our tolerance, and implementing the following rule:

$$|\sin(\theta_i - \theta_j)| > \alpha \Rightarrow \text{line } (i, j) \text{ fails.} \tag{17}$$

Alternatively, one could explicitly compute the flows:

$$F_{ij} = B_{ij} V_i V_j \sin(\theta_i - \theta_j), \tag{18}$$

which in the cases of the power flow and the third order models depend on the voltages V_i and V_j. However, this does not affect the results significantly, and using the angles as a criterion on whether a line fails or not, allows for direct comparison with the swing equation, which effectively also uses Supplementary Eq. (17) (multiplying both sides by the quantity B_{ij}).

We compare four different models, namely static and dynamic swing equation (see also main text), power flow and third order model, in the case of the five node network introduced in the main text in Supplementary Fig. 5. Note that the swing equation, reported in panel (c), and the third order power grid model in panels (e, f) return qualitatively similar results. The precise nature of the cascade differs and can also depend on the particular choice of the parameters used to extend the swing equation to the third order model. Nevertheless, a dynamical oscillatory transient is observed that leads to an overload in both cases. Similarly, the power flow equations reported in panel (d), return qualitatively similar results to those of the static swing equations in panel (b), while the observed values can differ quantitatively. Overall, analyses based on steady states, neglecting transient overloads, give very different results from those predicted by the dynamical models. Hence, in our article we used the simplest possible model available to capture the fundamentally dynamic nature of the power grid, namely the swing equation.

Validity of the swing equation

To overcome the short time scale validity of the swing equation, we considered a the third order model in the previois subsection. However, including voltage dynamics, while supposingly increasing the time of validity of the model [4], does not result in any qualitatively difference with respect to the (2nd order) swing equation in our simulations.
Even more so, we have found no evidence that including higher detail in the modeling would significantly change our results. For instance, Auer et al [21] have studied an even more detailed 4th order model. They have found that the 4th order model only differs asymptotically from the swing equation, while its transient dynamics on the time scale of seconds is very similar to that of the swing equation. This is because additional effects, like voltage dynamics, reactance differences etc., only enter when longer time scales (of minutes) are considered. Similarly, we do not expect the adoption of even more complicated models, like 6th order models, which include the sub-transient dynamics of the voltages [4], to drastically change the results either because we expect differences in the asymptotic and not the transient dynamics that is crucial for cascading events. This view is supported by a recent study [24] that also investigated cascading failures in power grids and found that static (DC) models underestimate the overall effect of cascades when compared to more detailed (AC) models.

We use the swing equation for periods of 10s of seconds, while it is typically stated that this equation is valid on the order of seconds [4, 12]. Firstly, our approach is justified by the fact that any alternative model mainly differs asymptotically and not in the transient dynamics from the swing equation. Second, the most relevant cascading events take place within the first few seconds in all our simulations. Inspecting Supplementary Fig. 5, we note that the initial failure takes place at $t_{\text{initial}} = 1$s and the final one at $t_{\text{final}} = 3 \pm 0.5$s, depending on the model and parameters. For larger networks of $N \sim 100$ nodes, like the Spanish grid, this time can slightly increase. Nevertheless, in most of our simulations, the majority of failures occurred within the first 5-10 seconds.

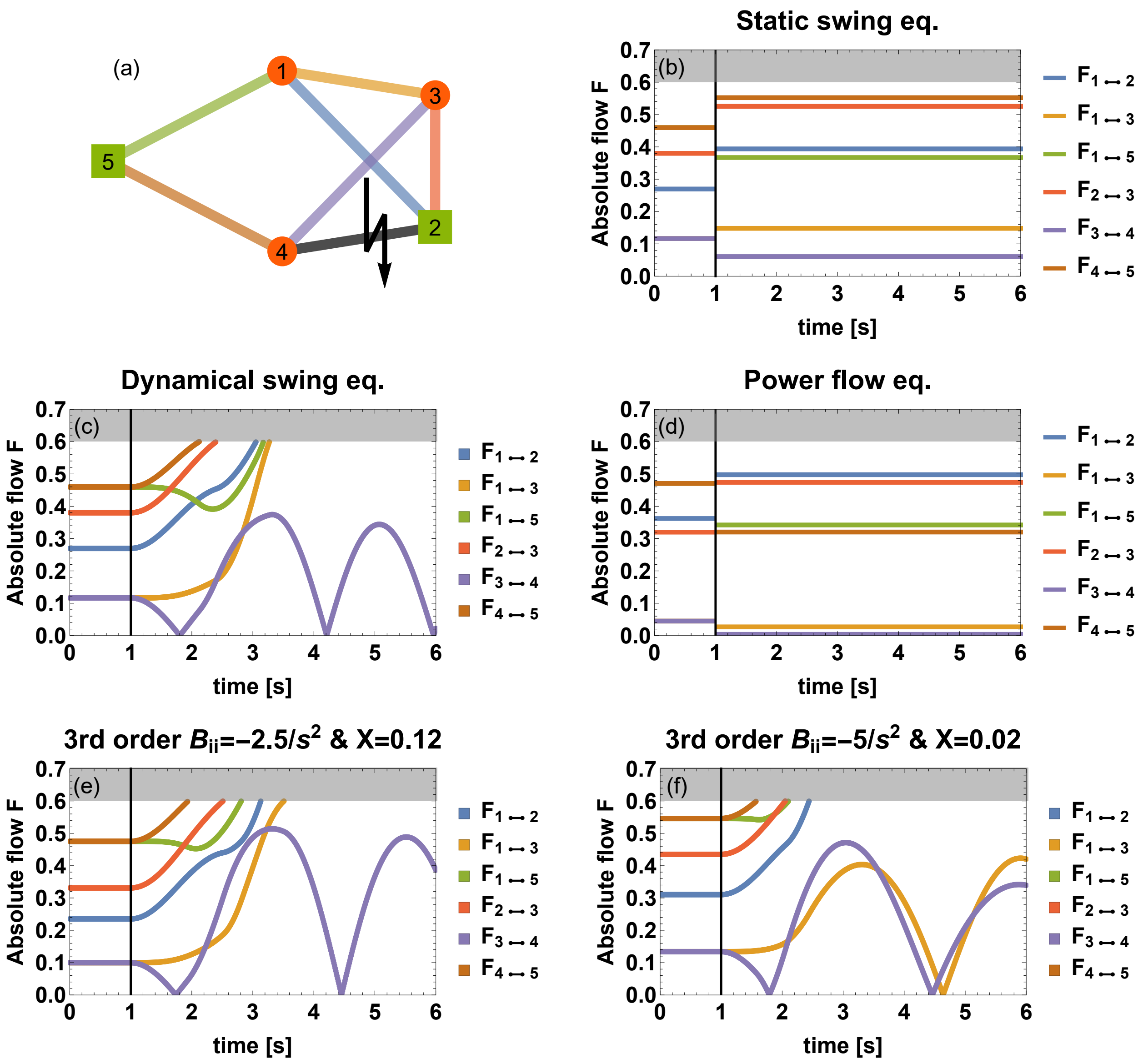

Supplementary Figure 5. Comparison of four different models. (a) The $N = 5$ node sample system used as a study case. (b)-(f) Plots of the time evolution of flows in the sample system when the triggering line indicated by the arrow is cut at time $t_{\text{trigger}} = 1\,s$. Other lines are assumed to fail when the flow reaches the gray area above $\alpha = 0.6$, which is our tolerance value. (b) Flows are based on the static swing equation, Supplementary Equation (3)-(4), i.e., the fixed point solution of the swing equation. (c) The dynamical swing equation, Supplementary Equation (1) is used for the flow calculation. (d) Flows are based on the power flow, Supplementary Equation (13) with $B_{ii} = -2/s^2$ on the diagonal and $G_{ij} = 4.5/s^2$ for all entries (including diagonal), and reactive power of the consumers $Q = -9.7/s^2$. (e)-(f) Flows are dynamically updated using the third order model, Supplementary Equation (16) with two different values of self-couplings B_{ii} and voltage droop X. The grid uses two generators $P^+ = 1.5/s^2$ and three consumers $P^- = -1/s^2$ and an susceptance of $B_{ij} \approx 1.63$ for non-diagonal elements. Qualitatively, static swing equation and (static) power flow equations return the same behavior. Similarly, third order models and dynamical swing equation display qualitatively the same behavior.

SUPPLEMENTARY NOTE 4

Predicting large cascades

It is crucial to avoid large scale blackouts. However, preventing them requires the identification of the critical triggering lines [2, 25]. In the main text, we presented a cascade predictor which is based on oscillations occurring during the transition from the old to a new fixed point after a line is removed. Here, we justify this assumption. Let us consider the 5-node sample system, displayed in Supplementary Fig. 5 (a). In Supplementary Fig. 6 we plot the flows of all lines, assuming that only the trigger line (marked with a lightning bolt in Supplementary Fig. 5 (a)) is initially cut and all other lines are left intact. Thereby, we exclude secondary failures as they are otherwise used in our cascading algorithm. Now, comparing Supplementary Fig. 6 with Supplementary Fig. 5 (c), where additional lines instead fail, we note that lines (2,3) and (4,5) get overloaded first because of their respective transient dynamics. However, Supplementary Fig. 6 reveals the oscillations around the new fixed point of the flows which is not visible in Supplementary Fig. 5 (c) because lines have failed when they exceed the maximum flow. Although the oscillations are not perfectly periodic, they are well approximated by damped sinusoidal functions. The flow based predictor proposed in the main text, which successfully identifies critical links, is based on these sinusoidal oscillations. See also Supplementary Fig. 4 for results for the British grid.

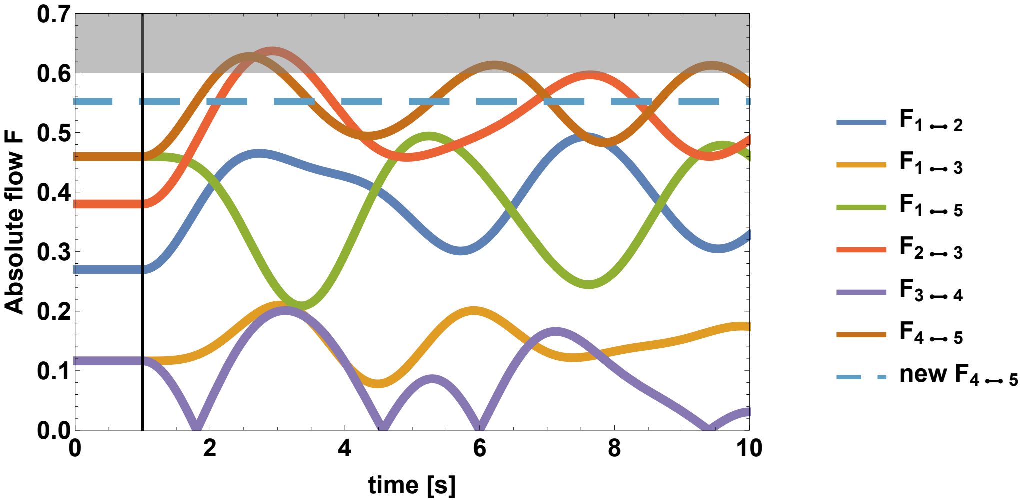

Supplementary Figure 6. The flows on all lines oscillate during the transition from the old to the new fixed point. Plotted are the absolute values of the flows in the five node sample system, when line (2,4) gets cut at time $t = 1$s and no further line overload is considered. Notice that lines (2,3) and (4,5) with the two largest flows at $t = 2$ seconds correspond to the first two lines that get overloaded in the full cascade algorithm. For illustration purposes, we included the new fixed point flows of line (4,5) as a dashed line. We observe oscillations of the flows approximately around their new fixed point flows which inspired the definition of the flow based cascade predictor.

SUPPLEMENTARY NOTE 5

Towards defining a propagation speed of cascades

Real world cascades often propagate through the power grid on a very fast time scale [1–3]. Is it possible to study such a propagation of the cascade in simulations based on the swing equation? In the main text, motivated by Ref. [26], we investigated this by introducing the measure of effective distance. We observed indeed a strong correlation between the time a cascade reaches a node and its effective distance from the initial trigger on the network, which might allow to define an average speed of the cascade propagation. To contrast the effective distance, we show here also the results obtained by using a standard graph distance to determine the speed: Assume two connected nodes i and j have distance $d_{ij} = 1/K_{ij}$ and distances over multiple edges are computed as shortest paths. For both graph distance and effective distance we compare the correlation coefficients as well as the slopes of the linear fit (pre-cursor of a propagation speed) of both approaches, see Supplementary Fig. 7. The linear fit using the standard graph distance [27] does not describe the data as well as it does in the case of the effective distance. Furthermore, when averaging over all potential trigger links, the distribution of the regression coefficient in the case of standard graph distance is centered at lower values of R and broader. This means that a linear relationship is stronger when the effective distance measure is adopted.

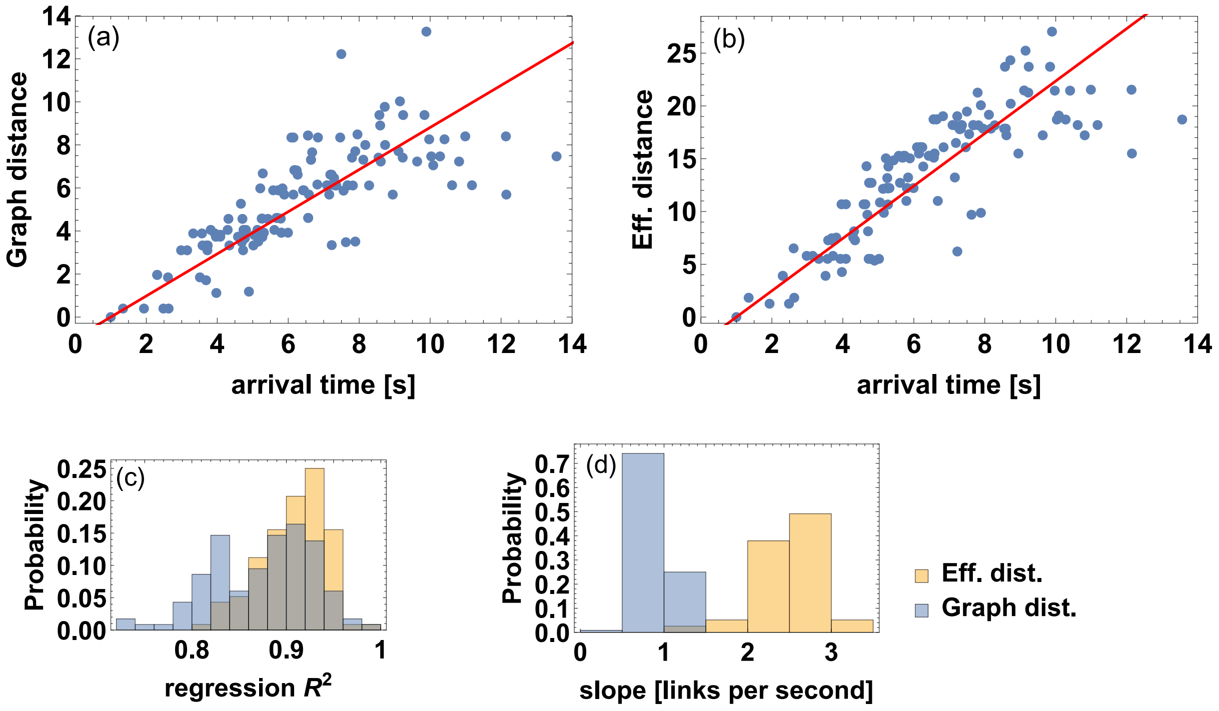

Supplementary Figure 7. The effective distance measure describes the constantly propagating cascade with a better linear relationship than the original graph distance. (a,b) Plotted are the distances of line failures with respect to the trigger line as a function of time for the Spanish grid with heterogeneous coupling. Every point in the plot corresponds to one line failure. The red line is a linear fit of the given points. We compare two different distance measures: (a) We use the original graph distance based on the weighted adjacency matrix, using $d_{ij} = 1/K_{ij}$. (b) We calculate the effective distance in the network based on [26]. (c,d) We record the squared regression R^2 and slope of the best linear fit for all lines with at least 10 line failures. (c) Effective distance provides a significantly better linear relation based on the regression coefficient. (d) The averaged slope is $\approx 2.55\,\text{links/s}$ for effective distance. We used the Spanish grid with distributed generators $P^+ = 1/s^2$ and heterogeneous coupling, as described above, and a tolerance of $\alpha = 0.55$.

SUPPLEMENTARY NOTE 6

Transient overload

So far, we assumed that a power line instantaneously trips when it is overloaded. However, this does not always have to be the case in real systems where, if the flow on a line exceeds the threshold only slightly, the line might not be cut for a short period of time [2, 3]. We show here that considering a non-instantaneous trip mechanism does not affect our results in a major way. More precisely, we investigate the case where line (i, j) fails if the flow F_{ij} exceeds the defined capacity C_{ij} for a time longer than a given certain allowed maximum overload time $t_{\text{max. overl.}}$. This is illustrated in Supplementary Fig. 8. Interestingly, the maximum transient overload time $t_{\text{max. overl.}}$ can be used to transition from our fully dynamical model to the static model by changing $t_{\text{max. overl.}}$ from zero, corresponding to the case of instant failures, to infinity, corresponding to a grid relaxing to a new fixed point before additional lines can fail. See also [11].

Typically, we observe that a moderate maximum transient overload time $t_{\text{max. overl.}} \in [0, 1]$s does not change our results significantly. Increasing $t_{\text{max. overl.}}$ results in fewer line cuts overall, so that events with a large number of line failures become less likely. However, qualitatively, our cascading results are not affected, as the changes are still small for $t_{\text{max. overl.}} \in [0, 1]$s. This is shown in Supplementary Fig. 9 that reports the probability for a certain amount of line failures for the Spanish grid with distributed power, i.e., equal number of generators and consumers, each with $P^+ = 1/s^2$ and $P^- = -1/s^2$, and homogeneous coupling with $K = 5/s^2$ throughout the grid.

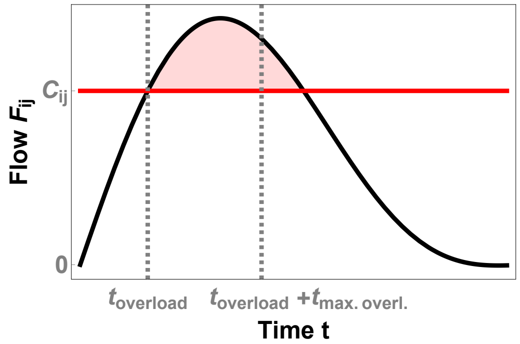

Supplementary Figure 8. Illustration of the introduction of a maximum transient overload time. The flow F_{ij} along line (i, j) (black curve) exceeds the capacity C_{ij} (red line) at time t_{overload} (first dashed line). If at time $t_{\text{overload}} + t_{\text{max. overl.}}$ (second dashed line) the flow is still above the capacity C_{ij}, the overloaded line (i, j) fails and it is removed from the network.

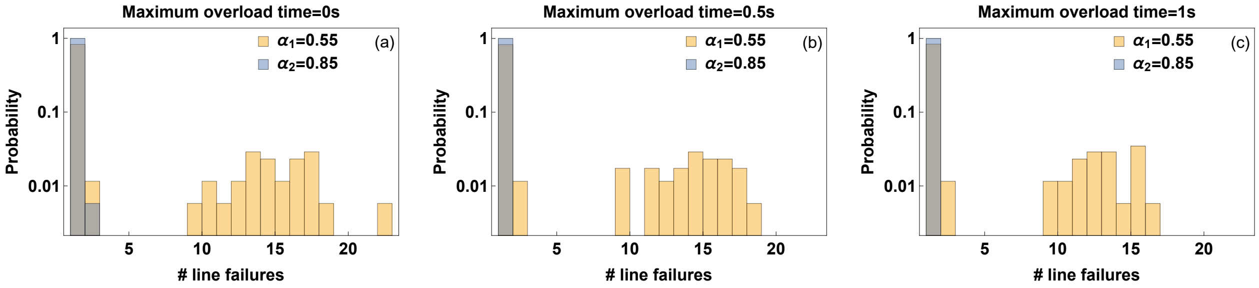

Supplementary Figure 9. Network damage distributions for the Spanish power grid in the case of different values of the maximum transient overload time $t_{\text{max. overl.}}$: (a) 0 seconds of overload, (b) 0.5 seconds of overload and (c) 1 second of overload is required to cause an overloaded line to fail. All panels use distributed power, i.e. equal number of generators and consumers, respectively with $P^+ = 1/s^2$ and $P^- = -1/s^2$, and homogeneous coupling with $K = 5/s^2$. For all plots we use two different values of tolerance α, where the lower one corresponds to the smallest simulated value (for $t_{\text{max. overl.}} = 0$) so that there are no initially overloaded lines ($N - 0$ stable). Increasing $t_{\text{max. overl.}}$ decreases the number of line failures but not drastically.

SUPPLEMENTARY REFERENCES

[1] Central Electricty Regulatory Commision (CERC). Report on the grid disturbances on 30th july and 31st july 2012. URL http://www.cercind.gov.in/2012/orders/Final_Report_Grid_Disturbance.pdf.

[2] Bundesnetzagentur für Elektrizität, Telekommunikation, Gas, Post und Eisenbahnen. Bericht über die Systemstörung im deutschen und europäischen Verbundsystem am 4. November. Tech. Rep., Technical report, German Federal Regulatory Agency for Electricity, Gas, Telecommunications, Postal and Railway Systems, Berlin, Germany (2006). URL https://www.bundesnetzagentur.de/SharedDocs/Downloads/DE/Sachgebiete/Energie/Unternehmen_Institutionen/Versorgungssicherheit/Berichte_Fallanalysen/Bericht_9.pdf?__blob=publicationFile&v=1.

[3] New York Independent System Operator. Interim report on the august 14, 2003, blackout (2004). URL https://www.hks.harvard.edu/hepg/Papers/NYISO.blackout.report.8.Jan.04.pdf.

[4] Machowski, J., Bialek, J. & Bumby, J. *Power system dynamics, stability and control* (John Wiley & Sons, New York, 2008).

[5] Filatrella, G., Nielsen, A. H. & Pedersen, N. F. Analysis of a power grid using a kuramoto-like model. *The European Physical Journal B* **61**, 485 (2008).

[6] Manik, D., Timme, M. & Witthaut, D. Cycle flows and multistability in oscillatory networks. *Chaos: An Interdisciplinary Journal of Nonlinear Science* **27**, 083123 (2017).

[7] Manik, D. *et al.* Supply networks: Instabilities without overload. *The European Physical Journal Special Topics* **223**, 2527 (2014).

[8] Wood, A. J., Wollenberg, B. F. & Sheblé, G. B. *Power Generation, Operation and Control* (John Wiley & Sons, New York, 2013).

[9] Crucitti, P., Latora, V. & Marchiori, M. Model for cascading failures in complex networks. *Physical Review E* **69**, 045104 (2004).

[10] Witthaut, D. & Timme, M. Nonlocal effects and countermeasures in cascading failures. *Physical Review E* **92**, 032809 (2015).

[11] Simonsen, I., Buzna, L., Peters, K., Bornholdt, S. & Helbing, D. Transient dynamics increasing network vulnerability to cascading failures. *Physical Review Letters* **100**, 218701 (2008).

[12] Kundur, P., Balu, N. J. & Lauby, M. G. *Power system stability and control*, vol. 7 (McGraw-hill New York, 1994).

[13] Wolfram Research Inc. Mathematica. Champaign, Illinois (2017).

[14] European Network of Transmission System Operators for Electricity (ENTSO-E). Statistical factsheet 2014. https://www.entsoe.eu/publications/major-publications/Pages/default.aspx. Accessed: 2015-09-01.

[15] Rosato, V., Bologna, S. & Tiriticco, F. Topological properties of high-voltage electrical transmission networks. *Electric Power Systems Research* **77**, 99–105 (2007).

[16] Rohden, M., Sorge, A., Timme, M. & Witthaut, D. Self-organized synchronization in decentralized power grids. *Physical Review Letters* **109**, 064101 (2012).

[17] Rohden, M., Sorge, A., Witthaut, D. & Timme, M. Impact of network topology on synchrony of oscillatory power grids. *Chaos* **24**, 013123 (2014).

[18] Ronellenfitsch, H., Manik, D., Horsch, J., Brown, T. & Witthaut, D. Dual theory of transmission line outages. *IEEE Transactions on Power Systems* **PP**, 1–1 (2017).

[19] Manik, D. *et al.* Network susceptibilities: Theory and applications. *Physical Review E* **95**, 012319 (2017).

[20] Schmietendorf, K., Peinke, J., Friedrich, R. & Kamps, O. Self-organized synchronization and voltage stability in networks of synchronous machines. *The European Physical Journal Special Topics* **223**, 2577–2592 (2014).

[21] Auer, S., Kleis, K., Schultz, P., Kurths, J. & Hellmann, F. The impact of model detail on power grid resilience measures. *The European Physical Journal Special Topics* **225**, 609–625 (2016).

[22] Ma, J., Sun, Y., Yuan, X., Kurths, J. & Zhan, M. Dynamics and collapse in a power system model with voltage variation: The damping effect. *PloS one* **11**, e0165943 (2016).

[23] Sharafutdinov, K., Matthiae, M., Faulwasser, T. & Witthaut, D. Rotor-angle versus voltage instability in the third-order model. *arXiv preprint arXiv:1706.06396* (2017).

[24] Cetinay, H., Soltan, S., Kuipers, F. A., Zussman, G. & Van Mieghem, P. Comparing the effects of failures in power grids under the ac and dc power flow models. *IEEE Transactions on Network Science and Engineering* (2017).

[25] Witthaut, D., Rohden, M., Zhang, X., Hallerberg, S. & Timme, M. Critical links and nonlocal rerouting in complex supply networks. *Physical Review Letters* **116**, 138701 (2016).

[26] Brockmann, D. & Helbing, D. The hidden geometry of complex, network-driven contagion phenomena. *Science* **342**, 1337–1342 (2013).

[27] Newman, M. *Networks: An Introduction* (Oxford University Press, Inc., New York, NY, USA, 2010).
