# Supplementary material for: Dynamically induced cascading failures in power grids
Source: Nat Commun. 2018 May 17;9:1975. doi: 10.1038/s41467-018-04287-5 (PMC5958123; doi:10.1038/s41467-018-04287-5)
Supplement: Supplementary file 3 — Description of Additional Supplementary Files [file 41467_2018_4287_MOESM3_ESM.pdf]

**Description of Additional Supplementary Files**

File Name: Supplementary Movie 1
Description: The movie illustrates the cascading dynamics for the five-node network discussed in Fig. 1 of the main text with two generators and three consumers. On the left, it displays the network with the edges indicating the instantaneous load (color coded). On the right, it shows the flows on each line. If the flow of a line exceeds the threshold, the respective line 'fails' and is removed from the network in the simulation.
